# Fermentation of Texturized Pea Protein in Combination with Proteases for Aroma Development in Meat Analogues

**DOI:** 10.1021/acs.jafc.3c08432

**Published:** 2024-02-23

**Authors:** Mónica Flores, Daniel Comes, Amparo Gamero, Carmela Belloch

**Affiliations:** † Institute of Agrochemistry and Food Technology (IATA)−Spanish Council for Scientific Research (CSIC), Agustín Escardino Avenue 7, 46980 Paterna, Valencia, Spain; ‡ Department of Preventive Medicine and Public Health, Food Science, Toxicology and Forensic Medicine, Faculty of Pharmacy, University of Valencia, 46010 Valencia, Valencia, Spain

**Keywords:** meat analogue, plant based, pea protein isolate, fermentation, off-flavor

## Abstract

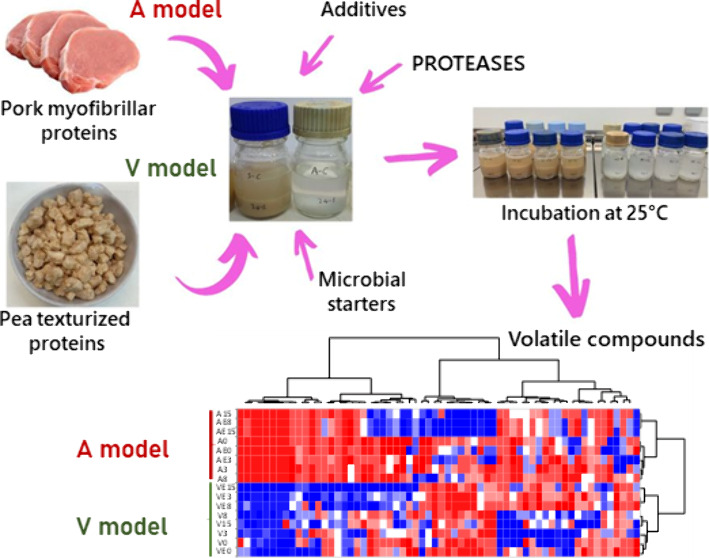

The potential use of texturized pea protein in meat analogues was investigated by comparing the effects of fermentation on pea and myofibrillar pork proteins in a model system including additives, microbial starters, and proteases. Model fermentation was controlled for 15 days by a pH decrease and microbial count and free amino acid increase. Besides, volatile production and sensory properties were evaluated at the end of fermentation. Protein type affected free amino acid generation and volatile profile. Models supplemented with proteases showed an increase in amino-acid-derived compounds (branched aldehydes and alcohols) and fruity odor notes. During fermentation, protease addition significantly reduced the production of linear aldehydes (pentanal, hexanal, and octanal) in vegetal models, while pyrazine compounds were not affected. This changes in the volatile profile reduced the legume beany odor but increased the perception of toasted cereal-like notes generated by the texturization process.

## 1 Introduction

Flavor is an essential issue in the development of meat and processed meat analogues.^1^ Changes in the ingredients or processing greatly affect the flavor of these products and, consequently, consumer preference, which is highly influenced by cultural habits and experience.^2^ The main components in the formulation of meat analogues are plant protein-rich ingredients, such as plant protein isolates and soy or wheat concentrates as well as legumes, like pea and lupine, rice, or potato.^3^ Peas belong to the Fabaceae family and are popular for their low cost and high protein content.^4^ The protein ingredients are the most important components for differentiation of meat analogues, because of their ability to provide a meat-like structure and nutritional health.^5^

Numerous studies have demonstrated that the inclusion of plant protein in food product formulation is the origin of undesirable volatile flavors.^6^ The removal or covering of the plant protein off-flavors as well as those originated from flavor interactions with the plant proteins are key in the study of meat analogue flavor.^7,8^ Indeed, many studies have focused on the flavor of cooked meat analogues and the effect of the addition of flavorings and aroma precursors during their manufacture.^9^ In the case of fermented and dry-cured products, aroma seems to be the biggest challenge for the formulation of meat analogues.

The fermentation of plant proteins has the potential to produce pleasant aroma compounds of interest in the design of fermented dry sausage analogues. The fermentation of plant-based foods to generate different flavor profiles is widely known in Asia since ancient times. Several of these fermented foods have been described as having a taste profile with umami characteristics. Moreover, many of these foods have been characterized in terms of their aroma profile and taste, as in the case of Chinese fermented soybean curd or white sufu and the Japanese fermented soybean paste miso.^10^

The protein sources most widely used in meat analogues are soy and pea isolates.^11^ However, the application of a fermentative process of these protein sources for production of fermented meat analogues has been scarcely investigated. Recent studies have proposed the fermentation of pea protein with a combination of lactic acid bacteria (LAB) and yeast starter cultures to reduce the off-flavors produced by the presence of hexanal and other oxidation products, like 2-pentylfuran, (*E*,*E*)-2,4-decadienal, hexanal, nonanal, (*E*,*E*)-2,4-nonadienal, octanal, (*E*)-2-nonenal, and (*E*)-2-octenal.^12,13^ Furthermore, the application of microbial consortia (LAB and molds) in the fermentative process in combination with enzymatic hydrolysis has been proposed as a way to improve the taste of soy protein isolates.^14^ This improvement was observed in both the taste and functionality (emulsifying and foaming properties) of the protein isolate, and in addition, the fermented protein isolates showed a reduced beany flavor.

In traditional fermented dry-cured products, flavor generation depends upon precursors produced during the fermentative process and the activity of microbial starters selected to ferment animal proteins. The ability of these starters to generate precursors and aromas has not been tested on plant proteins. Moreover, their activity may be hindered by their ability to hydrolyze vegetal proteins, which could be improved applying exogenous proteolytic enzymes. In summary, the aim of this study was to determine the functionality of microbial starters, combined with proteases, in the fermentation of texturized pea proteins. The fermentation process and its outcome were compared to that of an identical model system formulated with extracted pork meat proteins undergoing the same treatment. The results of this study could provide information about the potential use of texturized pea proteins in dry-cured meat analogue manufacturing.

## 2 Materials and Methods

### 2.1 Isolation of Myofibrilar Proteins from Pork Meat

The isolation of myofibrillar proteins was performed following the method of Molina and Toldrá^15^ using the muscle *longissimus thoracis et lumborum*. The process consisted of the homogenization of the meat with 0.03 N phosphate buffer at pH 7.4 using a stomacher (IUL masticator, Barcelona, Spain) followed by a centrifugation process at 10000*g* for 20 min. The pellet was collected, and the process was repeated 3 times for the removal of sarcoplasmic proteins. The final pellet was resuspended in a solution containing 0.1 N buffer phosphate, 0.7 M potassium iodide, and 0.02% sodium azide at pH 7.4, then filtered through glass wool, and diluted again in a solution containing 0.1 N buffer phosphate and 0.02% sodium azide at pH 7.4. Finally, the suspension was removed by centrifugation, and the pellet containing myofibrillar proteins was collected and used in the formulation of the models.

### 2.2 Preparation of Vegetal and Animal Fermentation Models

The fermentation model systems included animal or vegetal proteins together with common additives used in the fermentation of meat products (salt, glucose, and nitrifying agents), previously dissolved in distilled water and filter (0.22 μm) sterilized (Grynia, Labbox, Barcelona, Spain), and microbial starters. A commercial protease (Flavourzyme, >500 units/g, Sigma, Merck, Germany) was applied as a flavoring enzyme in some of the models, as described in Table S1 of the Supporting Information. Two models, animal (A) and vegetal (V), were prepared. The animal model (A) was formulated with the extracted myofibrillar proteins (8%, w/v), while the vegetal model (V) was prepared with texturized pea protein (8%, w/v, Manufacturas Ceylan, Valencia, Spain) previously homogenized in a blender. Two additional models containing the flavouring enzyme (0.02%) were prepared from the animal (AE) and vegetal (VE) models. The ingredients (3% NaCl, 2% glucose, 0.015% NaNO_2_, and 0.015% KNO_3_) of the four models were homogenized in a blender and inoculated with the commercial starter TRADI-302 (0.0125%), containing *Lactobacillus sakei*, *Staphylococcus carnosus*, and *Staphylococcus xylosus* (Chr. Hansen, Hoersholm, Denmark), and yeast *Debaryomyces hansenii* (L5, 10^6^ cells/mL),^16^ as indicated in Table S1 of the Supporting Information. The fermentation experiments of the four models (A, AE, V, and VE) were prepared in triplicate. The models were incubated at 25 °C in a heater (Incuterm Digit, Raypa, Barcelona, Spain), and samples were taken at days 0, 3, 8, and 15. The evolution of the fermentation was followed by the decrease in pH, microbial count, and free amino acid production. The sample for microbial analysis was homogenized with saline solution in a sampling bag with a side filter (Scharlab, Barcelona, Spain) using a Pulsifier II (3 pulses of 30 s, Microgen Bioproducts, Camberley, U.K.). The sample for physicochemical analysis was centrifuged at 10000*g* for 20 min, and the supernatant was filtered through a 0.2 μm filter (Minisart NML, Sartorius, Göttingen, Germany) and used for pH measurement with a portable pH meter (HI 99163, Hanna Intruments, Inc., Woonsocket, RI, U.S.A.). The supernatant was further used for free amino acid and volatile compound analysis. The samples for volatile analysis were acidified using 200 μL of tricloroacetic acid to inactivate protease activity, then neutralized with 1 N NaOH, and kept at −20 °C until further analysis. Additionally, at the end of the fermentation (15 days), the remaining fermented model was kept for sensory analysis.

### 2.3 Microbiological Analysis

The analysis was performed as described by Belloch et al.^17^ In summary, the homogenized samples were used to prepare decimal dilutions, which were subsequently spread in triplicates on the appropriate media plates for microbial counts as follows: total mesophilic bacteria (TMB) on plate count agar (PCA, Condalab, Madrid, Spain) at 30 °C for 2 days, LAB on De Man–Rogosa–Sharpe (MRS) agar (Scharlau, Barcelona, Spain) at 30 °C for 2 days, Gram-positive cocci (GC+) on mannitol salt agar (MSA, Scharlau, Barcelona, Spain) at 30 °C for 2 days, enterobacteria (E) on violet red bile glucose agar (VRBGA) at 37 °C for 24 h, and yeasts and molds (YM) on Rose Bengal agar chloramphenicol (Scharlau, Barcelona, Spain) at 30 °C for 3 days. Results from the microbial counts were expressed as log colony-forming units (CFU)/g.

### 2.4 Volatile Compound Analysis

Volatile compounds present in the headspace of the liquid sample were analyzed as described by Perea-Sanz et al.,^16^ by extracting the compounds with a solid-phase microextraction (SPME) device (Supelco, Bellefonte, PA, U.S.A.). Samples consisting of 4 mL of supernatant previously defrosted were placed in a headspace vial (20 mL, Gerstel, Germany) containing 1.88 g of NaCl and equilibrated at 37 °C for 30 min. Then, the volatile compounds were extracted for 1 h at 37 °C under shaking at 250 rpm using the SPME fiber (85 μm, CAR/PDMS StableFlex fiber, 1 cm). The extracted volatile compounds were analyzed in an Agilent HP 7890 series II gas chromatograph (GC) with a HP 5975C mass selective detector (Hewlett-Packard, Palo Alto, CA, U.S.A.) and a Gerstel MPS2 multipurpose sampler (Gerstel, Germany). The fiber was desorbed in the GC injection port at 240 °C for 5 min in splitless mode. Volatile compounds were separated using a DB-624 capillary column (30 m × 0.25 mm, 1.40 μm, Agilent Technologies, Santa Clara, CA, U.S.A.) and analyzed using the mass spectrometry (MS) detector in scan mode. Volatile compounds were identified by comparison to mass spectra from the library database (NIST’17), linear retention indices calculated using the series of *n*-alkanes C_8_–C_22_ (Aldrich, Merck, Germany),^18^ and comparison to authentic standards (Table S5 of the Supporting Information). Quantification was performed in scan mode using either total or extracted ion area (TIC or EIC) on an arbitrary scale. Each model supernatant was analyzed in triplicate; the results were expressed as abundance units (AU) × 10^–5^ per gram of protein in the media; and the differences in volatiles produced depending upon the protein source, animal or vegetal, were determined.

### 2.5 Free Amino Acid Analysis

The abundance of free amino acids released from the proteolytic activity in the liquid sample was measured following the methodology described by Aristoy and Toldrá,^19^ which includes the deproteinization and derivatization of the sample. Norleucine (10 mM in 0.01 M HCl) was used as an internal standard. The separation of free amino acids was performed by reversed-phase HPLC chromatography in an Agilent Series 1100 equipment (Agilent, Santa Clara, CA, U.S.A.) equipped with a Waters Nova Pack C18 column (3.9 × 300 mm, Waters Corporation, Milford, MA, U.S.A.) at 52 °C using a photodiode array detector.^20^ The separated amino acids were detected at 254 nm. Each medium supernatant was analyzed in triplicate. Identification of amino acids was achieved by comparison against a solution of mixed standards (Sigma, Merck, Germany), and quantification was based on the calculated response factors. They were calculated using five amino acid standard levels in the presence of the added internal standard (norleucine). The final results were expressed as milligrams of free amino acid per gram of protein in the model, and the differences in released free amino acids depending upon the protein source, animal or vegetal, were determined.

### 2.6 Sensory Analysis

The sensory analysis was performed from model samples at the end of the fermentation process (15 days) using the detection frequency method^21^ to reveal the aroma impact of volatile compounds in the models. Odors were evaluated by six trained panellists, four females and two males with an average of 40 years old, who evaluated the odors by smelling the model samples as reported by Belloch et al.^22^ The aroma descriptors were recorded, and the results were expressed as the number of times a descriptor was detected by the panellists.^21,23^

### 2.7 Statistical Analysis

Data were analyzed using the generalized linear model (GLM) procedure in the statistical software XLSTAT 2018 (Addinsoft, Barcelona, Spain). Data analysis, using the linear mixed model, included two factors: protein source (vegetal or animal) and enzyme as fixed effects and replicates as random effects. Differences between sample means were analyzed according to Tukey’s test, when a significant effect of the treatment group was detected (*p* < 0.05). Principal component analysis (PCA) was performed to evaluate the relationships between variables (pH, microbial counts, free amino acids, and volatiles) and models at the four sampling times. Heatmaps plotted using XLSTAT 2018 were based on the relative abundance of identified volatile compounds in the models at the four sampling times.

## 3 Results

The evolution of the fermentation, free amino acid content, changes in the volatile profile, and sensory analysis of the fermentative process in the vegetal and animal models, supplemented or not with a protease, was monitored. The analyses were performed at the beginning of the fermentation (day 0), at the middle (day 3 and 8), and at the end of the process (day 15).

### 3.1 Evolution of the Fermentation: pH and Microbial Counts

**Figure 1:**
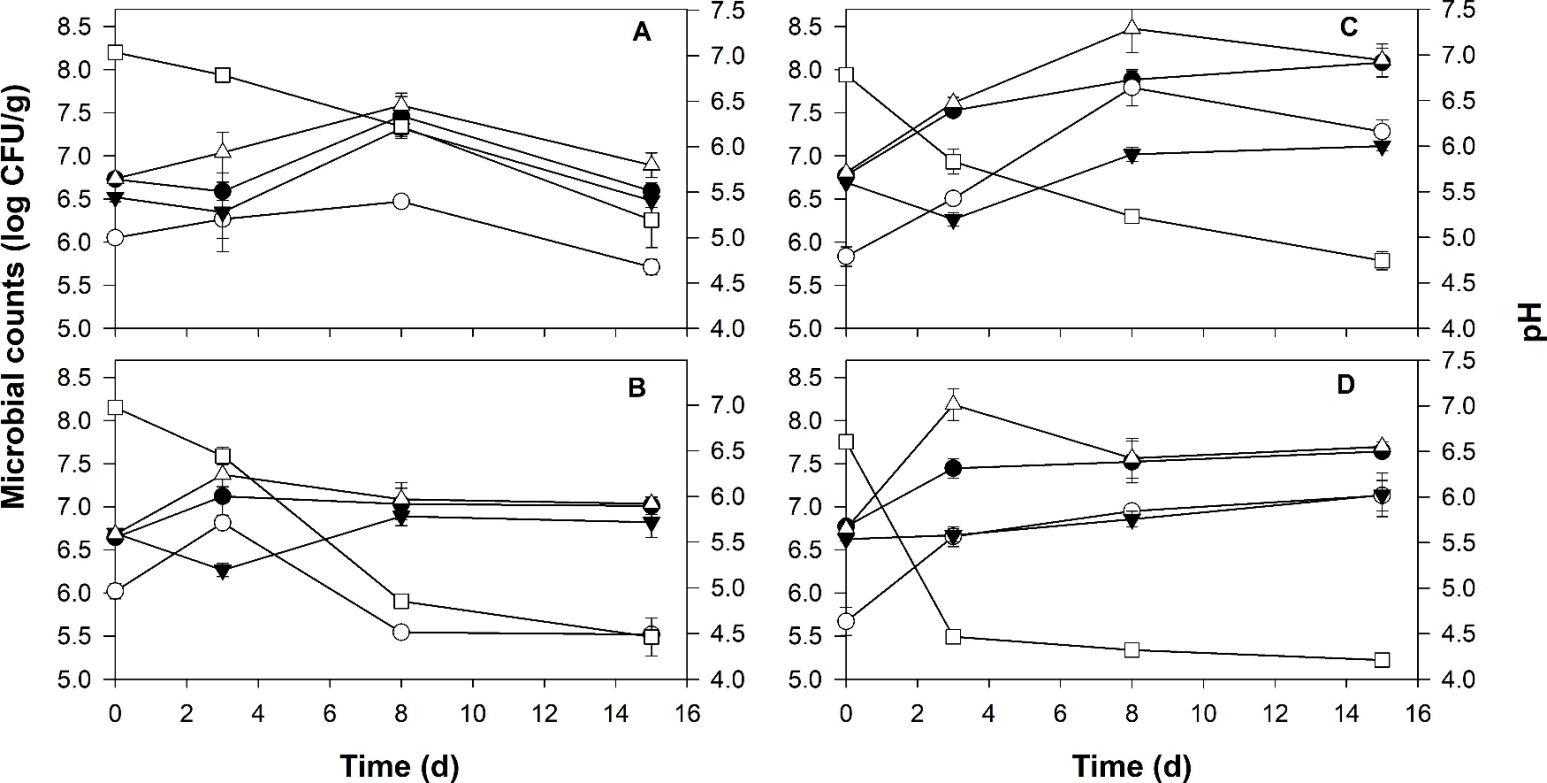
Effect of the fermentation time and addition of enzyme on pH and microbial counts (log CFU/g) of the vegetal and animal models. The results from the animal models are in panels A (without enzyme, A model) and B (with enzyme, AE model). The results from the vegetal models are in panels C (without enzyme, V model) and D (with enzyme, VE model). Symbols represent pH (□), TMB (△), LAB (●), GC+ (○), and YM (▼). Details about the individual variables and analysis of variance (ANOVA) results of the fermentation time and enzyme effects on the models are reported in Tables S2 and S3 of the Supporting Information.

The evolution of pH and microbial counts in the fermented models is shown in Figure 1 and Tables S2 and S3 of the Supporting Information. Values of pH decreased significantly during the fermentation of the animal (panels A and B of Figure 1) and vegetal (panels C and D of Figure 1) models. Moreover, the addition of the proteolytic enzyme significantly accelerated the pH decrease (Table S2 of the Supporting Information). The fermentation time significantly increased microbial counts, usually at days 3 or 8 of fermentation. Microbial counts were lower in the animal model (Figure 1A) than the vegetal model (Figure 1C). In contrast, the addition of the protease decreased bacterial counts in both models (panels B and D of Figure 1 and Table S3 of the Supporting Information). This decrease was significant in the case of GC+ counts in the animal model (AE), while in the vegetal model (VE), the effect was observed in both GC+ and LAB counts. In contrast, the differences in PCA and YM counts between models with or without enzyme were not significant. No enterobacteria were detected along the fermentation process.

### 3.2 Determination of Free Amino Acids in the Models along the Fermentation

**Figure 2:**
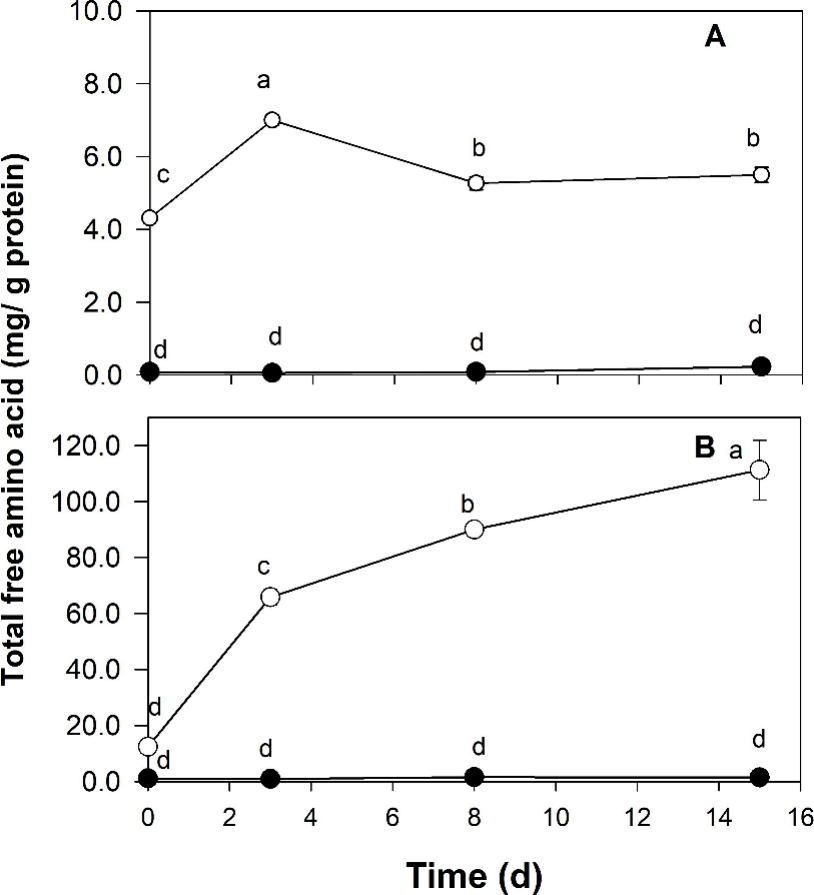
Evolution of the total free amino acid content (mg/g of protein) in animal and vegetal models. Panels: A, animal model without enzyme (A, ●) and animal model with enzyme (AE, ○); B, vegetal model without enzyme (V, ●) and vegetal model with enzyme (VE, ○).

The total content of free amino acids along the fermentation is reported in Figure 2, while the values for individual amino acids are in Tables S4 and S5 of the Supporting Information. In general, the free amino acid content significantly increased in all models along the fermentation time (Figure 2), except for a few amino acids (Glu, His, Thr, Met, Phe, and Trp) in the animal models (Table S4 of the Supporting Information). The addition of enzyme also significantly increased the amino acid content in both vegetal and animal models (Tables S4 and S5 of the Supporting Information). This increase was about 100 times higher in the vegetal models than the animal models (Figure 2). In the animal model, the addition of enzyme significantly increased the production of amino acids Ala, Pro, Val, Ile, Leu, Orn, and Lys, but the amount produced was only 2–3 times higher than the initial time. In contrast, the amount of free amino acids produced by enzyme addition in the vegetal model was higher, around 8-fold in the case of Try, Ala, Thr, and Glu and 12-fold in the case of Phe, Ile, Leu, and Val.

### 3.3 Differences in the Volatile Profile between the Models along the Fermentation

**Figure 3:**
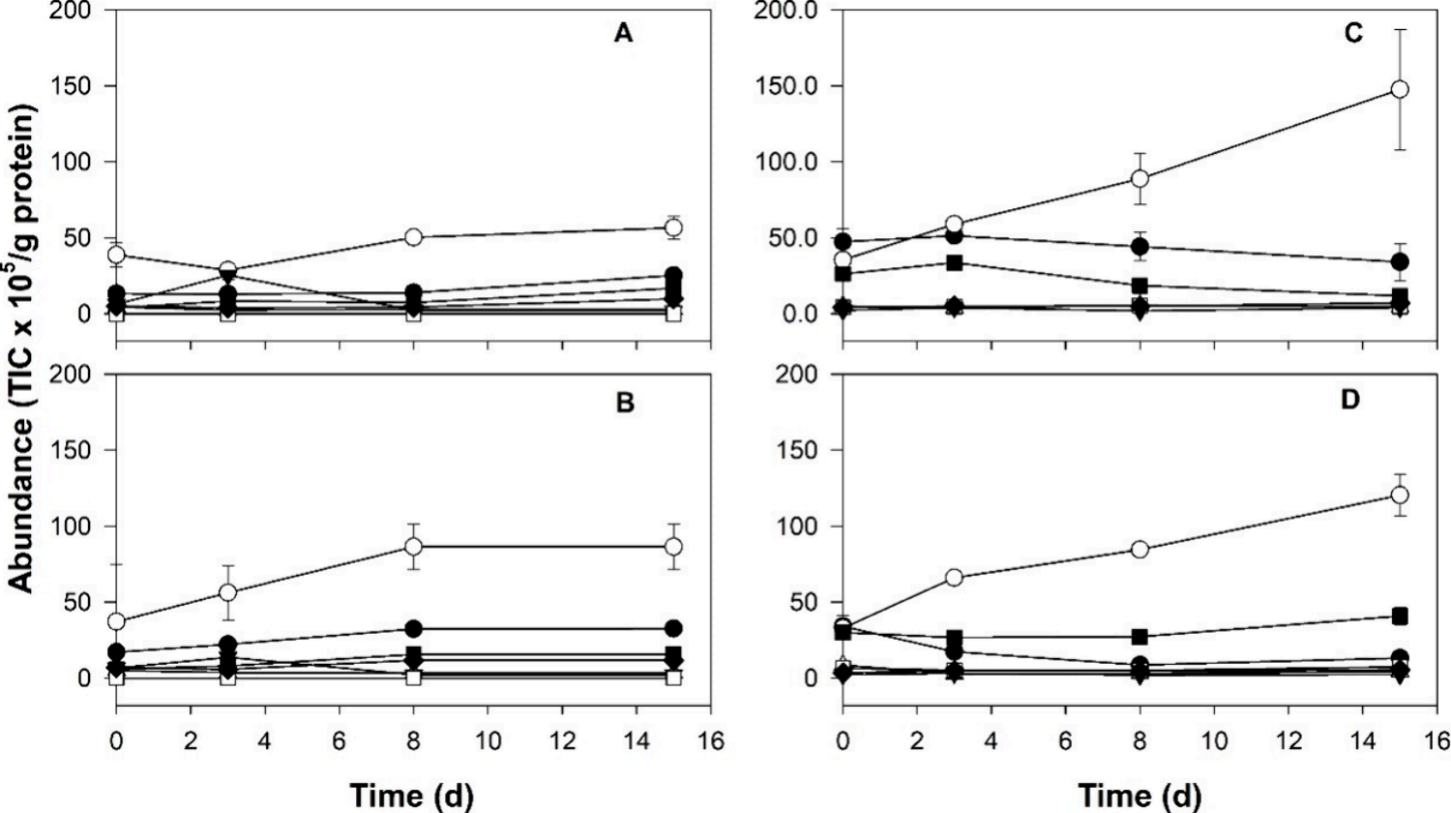
Abundance (AU × 10^5^/g of protein) of volatile compounds summarized by chemical group detected in the headspace of the animal and vegetal models along the fermentation. Panels: A (animal model without enzyme, A), B (animal model with enzyme, AE), C (vegetal model without enzyme, V), and D (vegetal model with enzyme, VE). Compounds: aldehydes (●), alcohols (○), esters (▼), alkanes (△), ketones (■), pyrazines (□), and other (◇).

**Figure 4:**
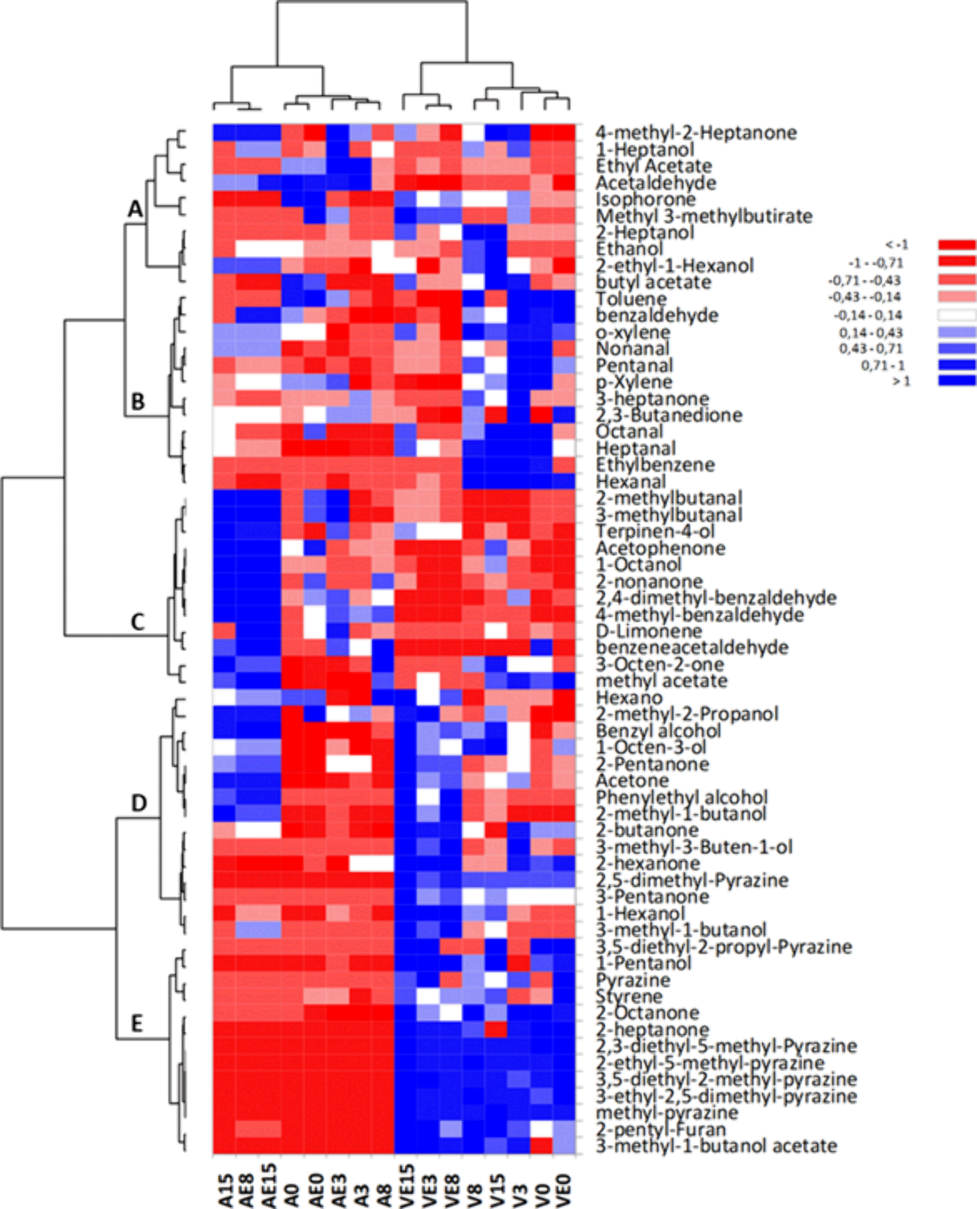
Heatmap representing the volatile profile of the animal and vegetal models during the fermentation. Samples: animal models without (A) and with (AE) enzyme and vegetal models without (V) and with (VE) enzyme. Numbers in the samples represent fermentation time in days. Colors in the heatmap indicate the relative abundance of each volatile compound: blue, relatively high abundance; red, relatively low abundance.

The volatile organic compound (VOC) profile was very different in the vegetal and animal models (Figures 3 and 4 and Tables S6–S8 of the Supporting Information). A total of 62 VOCs were identified in the headspace of the model, but the chemical structure was confirmed in only 54 of them (Table S6 of the Supporting Information). A total of 8 VOCs, including 3 pyrazines, were tentatively identified by mass spectrometry. The main difference between the vegetal and animal models was the presence of pyrazines in the vegetal models, which were absent in the animal models. Also, four additional compounds, 3-methyl-3-buten-1-ol, 3-methyl-1-butanol acetate, ethylbenzene, and 3-pentanone, were only detected in the vegetal models (Table S6 of the Supporting Information).

The evolution of the VOC profile classified by chemical group (Figure 3) along the fermentation of all models indicates that alcohols constituted the most abundant group, followed by aldehydes and ketones. The evolution of the fermentation can be recognized by the significant increase of alcohols with time in all models (Figure 3), with this increase being higher in the vegetal model (Figure 3C) than the animal model (Figure 3A). Besides, the addition of enzyme impacted the vegetal and animal models differently. In the vegetal models (Table S8 of the Supporting Information), alcohols, such as ethanol and 2-ethyl-1-hexanol, were the most abundant compounds found in the V model, while in the VE model, several methyl-branched alcohols (2- and 3-methyl-1-butanol) and phenylethyl alcohol increased. Similarly, branched aldehydes 2-methyl and 3-methyl butanal were in higher abundance in the VE model than the V model. The abundance of ketone compounds generally increased in the VE model with respect to the V model (Figure 3D). Few changes were observed in pyrazine abundance along the fermentation, and the addition of enzyme did not produce a clear trend. In contrast, the addition of enzyme in the animal model did not cause many significant differences in the volatile profile (Table S7 of the Supporting Information). The main differences were the increase in branched aldehydes (2-methyl and 3-methyl butanal) in the AE model, as happened in the vegetal model VE (panels A and B of Figure 3 and Table S7 of the Supporting Information).

A more comprehensive comparison of the compounds constituting the volatile profile of the models was plotted in a heatmap with hierarchical clustering (Figure 4). The dendrogram at the top shows that the models are divided in two groups by the type of protein employed, animal (left) versus vegetal (right). Moreover, differences within each group can also be observed. In the vegetal model, the effect of the enzyme had a larger impact than the fermentation time, as samples VE3, VE8, and VE15 appear separated from the rest of the samples. In the animal model, the main impact was caused by the fermentation time, as samples A15, AE8, and AE15 were separated (left) from the rest. The dendrogram on the left shows which compounds support the differences between and within the models. The presence of pyrazines and a few ketones constitute the core of cluster E, which separates between the vegetal and animal models. The remaining clusters of compounds account for the main differences within the models. Cluster D composed of several alcohols and ketones separates samples A8, A15, and AE15 from the other samples in the animal model as well as the VE samples from the V samples in the vegetal model. The separation of A8, A15, and AE15 samples is also supported by compounds in cluster C, composed of several ketones, branched aldehydes, and alcohols. Finally, cluster B constituted mainly by linear aldehydes separates initial samples 0 and 3 from later samples 8 and 15 in the V model.

**Figure 5:**
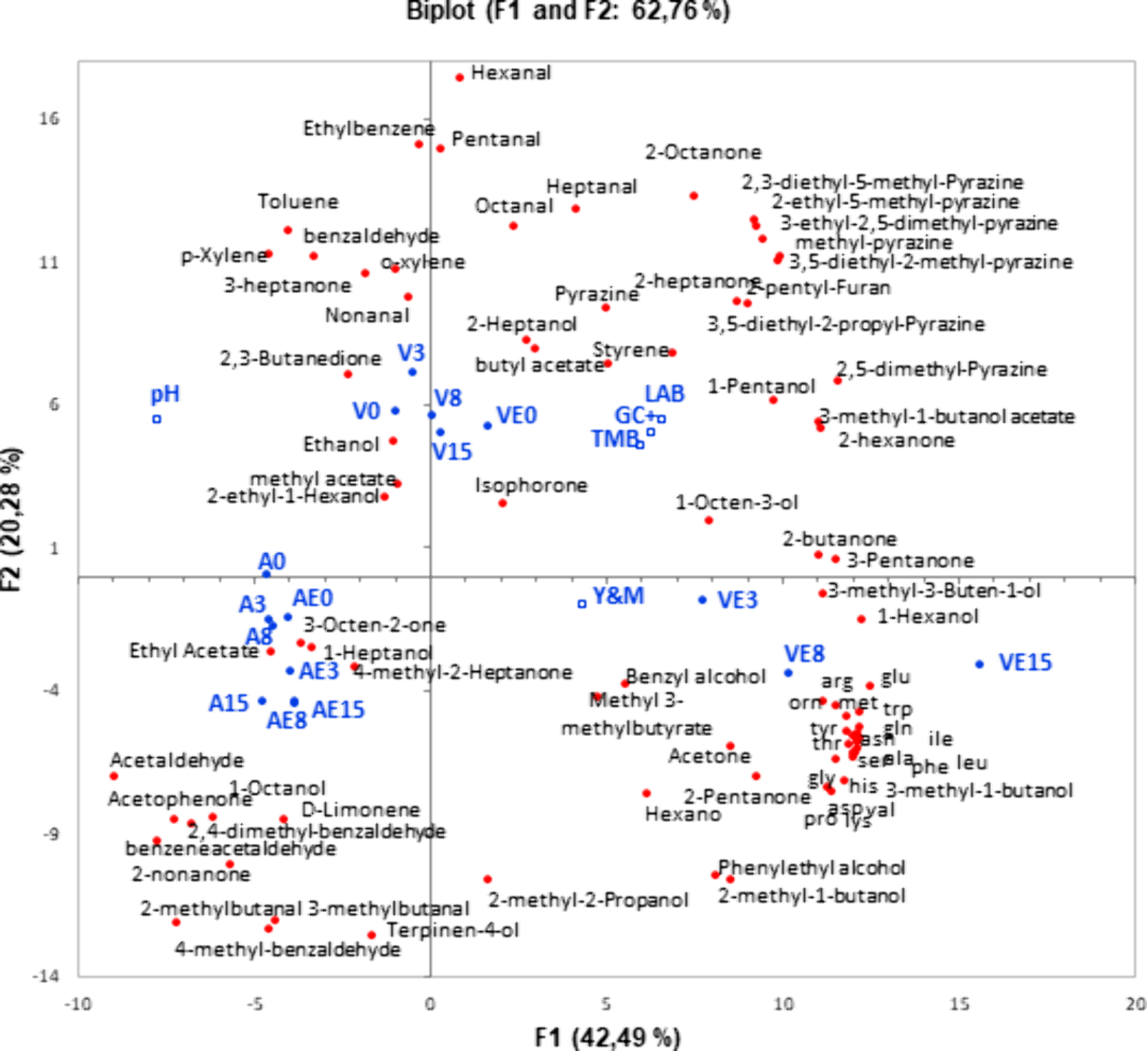
PCA showing the relationship among variables, microbial counts, pH, free amino acids, and volatile compounds and the animal and vegetal models along the fermentation. Animal models are represented by samples A (without enzyme) and AE (with enzyme), whereas vegetal models are represented by samples V (without enzyme) and VE (with enzyme). The numbers in the models represent the fermentation time in days of the samples.

A further analysis of the data was applied to study the effect of the time and enzyme addition on the fermentation of pea protein versus pork myofibrillar protein, and the results were plotted in a principal component analysis (PCA) (Figure 5). The PCA explained 62.8% of the variability. The first factor (42.5%) separated the animal samples from the vegetal samples, whereas the second factor (20.3%) separated the samples containing enzyme by fermentation time. Microbial counts and free amino acids were clearly related to the V model samples. Moreover, it is worth noting that all free amino acids are closely related to the VE model samples. With regard to the volatile compounds, pyrazines seem to be the main variable separating V models from A models, while alcohols and aldehydes separate the VE model from the V model.

### 3.4 Sensory Properties of the Fermented Models

**Figure 6:**
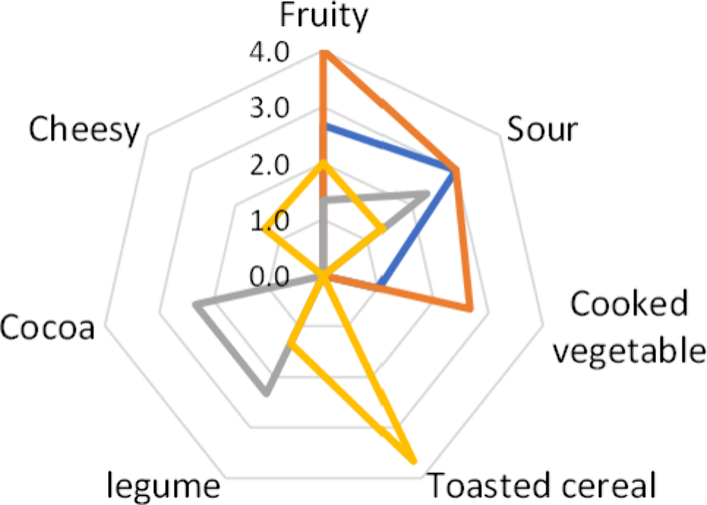
Odor profile of the animal and vegetal models after 15 days of incubation. Animal models are represented by A (blue) and AE (orange) lines, whereas vegetal models are represented by V (gray) and VE (yellow) lines.

The odor profile of the models was evaluated at the end of the fermentation time (15 days) (Figure 6), and significant (*p* < 0.05) differences were found among all models. The animal models were defined by descriptors fruity, sour, and cooked vegetal, while the vegetal models were described by toasted cereal, legume, cocoa, and cheesy odor notes in addition to fruity and sour. The addition of enzyme had a significant impact on the odor profile of the vegetal model. The legume and cocoa notes in the V model were replaced by toasted cereal, cheesy, and fruity notes in the VE model. Furthermore, the addition of enzyme significantly decreased the sour odors. In the case of the animal models, the addition of enzyme (AE model) only increased the fruity and cooked vegetal odors already present in the A model.

## 4 Discussion

To develop attractive plant-based fermented meat analogues, we have evaluated the potential of pea protein isolate fermentation in combination with enzymatic proteolysis to improve flavor. Moreover, we have compared these findings to those obtained applying a similar fermentative process using extracted meat proteins. The results from our study show (Figure 1) that the fermentation process progressed in a similar way using texturized pea protein or myofibrillar pork proteins, although the presence of the enzyme (protease) accelerated the process. This may be due to the increase in free amino acids produced by the proteolytic activity (Figure 2), which would increase the metabolic activity of LAB and, consequently, the decrease in pH. Enzyme addition (VE and AE models) caused a slightly negative effect on LAB counts in both models; however, this decrease did not seem to have a large impact in either the pH decrease or the fermentation progress. On the contrary, yeast growth was not affected by the presence of enzyme in the models, which could have important consequences for aroma generation.^24^ The most important difference between the models, animal (A) and vegetal (V), was the higher microbial counts in the vegetal model, which might indicate that the texturized pea protein is more accessible to the microorganisms, thus facilitating proteolysis activity. This agrees with the slight increase in free amino acid abundance in the V model with respect to the A model (Figure 2 and Tables S4 and S5 of the Supporting Information). Previous studies have demonstrated that hydrolysis of myofibrillar proteins using *Staphylococcus carnosus* exoproteases highly increases the concentration of free amino acids Glu and Gly and moderately increases the concentration in the case of His, Thr, Val, Leu, Phe, and Lys.^25,26^ In contrast, the addition of the starter culture (A model), which also includes *S. carnosus*, did not produce a significant increase of protein hydrolysis, and only the addition of the commercial protease (AE model) produced a significant increase of the proteolytic activity against myofibrillar pork proteins. In agreement with previous studies, some of the most abundant free amino acids produced in the AE model (animal model with enzyme) (Table S4 of the Supporting Information) were the same as those produced by hydrolysis of myofibrillar proteins using *S. carnosus* exoproteases.^26^ The texturized pea protein (VE model) underwent a similar proteolysis and fermentative process to the animal model (AE), but in comparison, the free amino acid yield in the former was significantly higher than that in the latter. This result would indicate that the pea protein is more accessible to enzymatic activity than the myofibrillar pork proteins. Moreover, the large proteolysis yield of the vegetal model (VE) would suggest that exopeptidase activities are present. Furthermore, the amino acid composition of plant proteins can be very different from that found in meat proteins^26^ and, in the case of pea proteins, the most abundant amino acids are Glu, Arg, Leu, and Lys, whereas the less abundant amino acids are Met and Cys, in agreement with previous studies.^27^

The generation of free amino acids is closely related to the formation of volatile compounds affecting aroma. For example, in fermented meat products, the generation of sulfur amino acids promotes the formation of sulfur compounds, which contribute to savory properties of the meat product.^28^ An important result from our study was that the fermented models, animal and vegetal, generated different volatile profiles, which were derived from the different amino acid composition of the proteins present in the models. The volatile profile of hydrolyzed myofibrillar proteins using *S. carnosus* exoproteases^26^ has been reported to include VOCs, such as linear aldehydes, alcohols, and ester compounds, after only 2 h of hydrolysis. Among these compounds, two were found derived from phenylalanine: benzenacetaldehyde and phenylethyl alcohol. The generation of these two compounds was also observed in the animal models (A and AE) (Table S7 of the Supporting Information). However, benzeneacetaldehyde was absent or scarcely produced in the vegetal models (V and VE), whereas phenylethyl alcohol was abundantly found in the VE model (Table S8 of the Supporting Information). The main differences between a purely enzymatic hydrolysis^26^ and our study are the addition of microbial starters and the longer incubation times (up to 15 days). These differences were responsible in the VE model for the generation of compounds derived from phenylalanine (benzenacetaldehyde and phenylethyl alcohol) as well as those derived from isoleucine and leucine, like branched aldehydes (2-methyl- and 3-methyl butanal) and their respective alcohols (Figure 4).

The effect of the long fermentation time, applied in our models, on the volatile profile is not easy to analyze because from the beginning of fermentation (day 0) both models, animal and vegetal, had a very dissimilar volatile profile. The largest difference was the presence in the pea protein models of odor-active carbonyl compounds (linear aldehydes and 2-pentyl furan; Figure 4) responsible for the beany flavor^29^ and pyrazine compounds derived from the degradation of fatty acids and amino acids, respectively.^30^ The presence of different aldehydes, ketones, and pyrazines responsible for the beany flavor in the vegetal models largely depends upon not only the initial pea protein composition^31^ and texturization process^29^ but also the volatile extraction technique employed during analysis, which affects the VOC profile qualitatively and quantitatively.^32^ The large influence of these factors on the VOC profile limits comparisons of results between studies using the same extraction conditions. Nevertheless, odor compounds responsible for the pea protein isolate flavor, such as hexanal, benzaldehyde, heptanal, and 1-octen-3-ol, derived from lipid oxidation processes^33,34^ were also present in the vegetal models (Figure 4). With regard to the pyrazines, those present in vegetal models may be derived from Maillard reactions during the texturization process as 2,5-dimethyl-pyrazine.^30^ Other pyrazines are inherent constituents of the pea protein as methoxypyrazines,^35^ while 2-isobutyl-3-hydroxypyrazine varies during the isolation process of pea proteins and affects the aroma profile.^34^

The contribution of microbial starters to food aroma has been widely explored.^36^ Moreover, their application in fermented meat products for their ability to transform free amino acids, generated by the endogenous proteolytic system, into volatile compounds has been amply proven.^37^ In the case of vegetal proteins, most efforts have focused on the removal of beany off-flavors, especially on the transformation of aldehydes and ketones into alcohols or carboxylic acids by the activity of alcohol dehydrogenase (ADH) and aldehyde dehydrogenase (ALDH) present in microorganisms.^31^ Among the most studied microorganisms for this application are LAB (*Lactobacillus acidophilus*, *Limosilactobacillus fermentum*, *Lactiplantibacillus plantarum*, and *Streptococcus thermophilus*) and *Saccharomyces cerevisiae*. In the case of the animal and vegetal models used in our study, both the formulation of the models and the microbial starter were selected to imitate a fermented meat product; therefore, bacterial (TRADI-302, Chr. Hansen, Denmark) and fungal starters^16^ used for that purpose were applied.

Since the beginning of the fermentation, ketones and aldehydes were detected in high abundance in the vegetal models (V and VE) (Figure 4), as already observed in previous studies.^13^ Fermentation reduced aldehydes, such as pentanal and nonanal in the V model and hexanal in VE model. Similar reductions of hexanal and nonanal have been attributed to *S. cerevisiae* and *L. plantarum* fermentations of pea protein for 6 and 8 h, respectively.^13^ However, fermentation was not able to reduce ketones and pyrazines, especially 2,5-dimethylpyrazine, which contributes to the nutty and cereal-like odor in fermented pea.^30^ The alcohol increase observed during fermentation of the vegetal models is in accordance with previous studies.^13^ The presence in the VE model of methyl-branched alcohols (2-methyl and 3-methyl butanol) (Figure 4 and Table S8 of the Supporting Information) could be a direct consequence of the large amounts of free amino acids, which made the generation of methyl-branched alcohols possible by microbial activity.

The impact of the VOCs on the aroma of the fermented models cannot be solely determined by the calculation of the odor activity values (OAVs). Besides, the extraction method employed (SPME) only allows for the comparison of the volatile profile among models and fermentation time and requires the application of accurate quantitation methodologies.^38^ These limitations were overcome applying a sensory analysis of the models. This analysis revealed that fruitiness and cooked vegetal odors detected in the AE model could be explained by the presence of d-limonene, benzene, acetaldehyde, branched aldehydes, and terpinen-4-ol, respectively (Figure 5). In the vegetal model (VE), the reduction of the legume and cocoa odor notes as well as the increase of toasted cereal notes was related to the reduction of aldehydes.^29,31^ On the contrary, pyrazine abundance was not affected by the fermentation time or enzyme addition and probably increased the perception of the nutty and cereal-like odor in the vegetal models.^30^ In this regard, recent studies have revealed the potential of plant hydrolysates to simulate the meaty aroma by producing volatile compounds through Maillard reactions.^39,40^ The combination of Maillard reactions and protein hydrolysis, using the same enzyme applied in our study, on wheat and rice^40^ and soy^39^ revealed similar nutty and toasted aroma notes. These odors were attributed to alkyl pyrazines resulting from the Maillard reaction and derived from the free amino acids generated thorough hydrolysis. Similarly, in our study, the presence of alkyl pyrazines was detected at the beginning of the fermentation; therefore, their origin can be attributed mainly to the texturization process of pea proteins, which employed a high pressure and temperatures.^29^

In conclusion, the potential of the fermented vegetal models to simulate the meaty aroma should be focused on the elimination of not only the beany compounds but also the pyrazines producing toasted cereal-like odors. Moreover, the generation of volatiles, which could reduce or mask these off-aromas in the vegetal model, is largely affected by the level of proteolysis and generation of free amino acids, which are used as volatile precursors by the microbial starters. Finally, the whole food matrix composition and not only the proteins is a source of flavor compounds; therefore, the interaction mechanisms between proteins, fat, and volatile compounds will affect flavor perception in plant-based foods.^35^ In summary, these models are far from a real food system, and the elucidation of the aroma impact of the compounds generated through fermentation of plant proteins should be performed through proper quantitation on future developed plant-based foods.

## Supporting Information Available

Supplementary tables with the composition of animal and vegetal models, identification of volatile compounds, and data of microbial counts, free amino acid content, and volatile compound content in the models (PDF)

## Abbreviations Used

SPME: solid-phase microextraction
TMB: total mesophilic bacteria
LAB: lactic acid bacteria
GC+: Gram-positive cocci
E: enterobacteria
YM: yeasts and molds
VOC: volatile organic compound
OAV: odor activity value
