# Supplementary material for: Fermentation of Texturized Pea Protein in Combination with Proteases for Aroma Development in Meat Analogues
Source: J Agric Food Chem. 2024 Feb 23;72(9):4897–905. doi: 10.1021/acs.jafc.3c08432 (PMC10921465; doi:10.1021/acs.jafc.3c08432)
Supplement: Supplementary file 1 — jf3c08432_si_001.pdf [file jf3c08432_si_001.pdf]

Supplementary Tables

Manuscript title: Fermentation of texturized pea protein in combination with proteases for aroma development in meat analogues

Authors: Mónica Flores[1]*, Daniel Comes[1], Amparo Gamero[2], Carmela Belloch[1]

[1]Institute of Agrochemistry and Food Technology (IATA-CSIC), Agustín Escardino Avenue 7, 46980, Paterna (Valencia), Spain.

[2]Department of Preventive Medicine and Public Health, Food Science, Toxicology and Forensic Medicine, Faculty of Pharmacy, University of Valencia, Spain.

*Corresponding author M. Flores. E-mail address: mflores@iata.csic.es

Table S1. Composition of animal (myofibrillar pork protein) and vegetal (texturized pea protein) models.

	Models			
**Composition (g/100ml)**	**Animal<br>A**	**Animal +<br>Enzyme<br>AE**	**Vegetal<br>V**	**Vegetal +<br>Enzyme<br>VE**
**Myofibrillar pork protein**	8	8	-	-
**Texturized pea protein**			8	8
**NaCl**	3	3	3	3
**Glucose**	2	2	2	2
**$NaNO_2$**	0.015	0.015	0.015	0.015
**KNO_3**	0.015	0.015	0.015	0.015
**Starter TRADI-302**	0.0125	0.0125	0.0125	0.0125
**D. hansenii (L5) (cells/ml)**	10^6	10^6	10^6	10^6
**Protease (Flavourzyme) (g/g protein)**		0.02		0.02

Table S2. Microbial counts (log cfu/g) and pH from the fermentative process of the animal models without and with proteolytic enzyme. Samples were taken at 0, 3, 8 and 15 days of incubation.

	Animal model without enzyme								Animal model with enzyme											
	**A0[1]**		**A3**		**A8**		**A15**		**AE0**		**AE3**		**AE8**		**AE15**		**RMSE[2]**	P_t[3]	P_E	P_{txE}
**PCA**	6.74	bc	7.04	abc	7.59	a	6.89	abc	6.68	c	7.4	ab	7.09	abc	7.04	abc	0.24	**	ns	ns
**LAB**	6.73	b	6.59	b	7.46	a	6.60	b	6.64	b	7.12	ab	7.03	ab	7.01	ab	0.24	**	ns	**
**GC+**	6.05	bcd	6.27	bc	6.47	ab	5.71	de	6.02	cd	6.81	a	5.54	e	5.52	e	0.15	***	*	***
**YM**	6.52	ab	6.35	c	7.32	a	6.48	ab	6.69	ab	6.27	b	6.89	ab	6.82	ab	0.31	**	ns	*
**pH**	7.04	a	6.79	ab	6.22	b	5.19	c	6.97	a	6.44	ab	4.85	cd	4.46	d	0.23	***	***	***

[1]Animal models containing myofibrillar proteins without (A) and with (AE) enzyme at 0, 3, 8 and 15 d of incubation. [2]RMSE: root mean square of the errors. [3]*Pf*: *P* value of the time effect, P_E: *P* value of enzyme effect, P_{txE}: *P* value of interaction between time and enzyme effects. ***: $P < 0.001$; **: $P < 0.01$; *: $P < 0.5$; ns: $P > 0.05$. [4]Different letters in the same row indicate significant differences among models and sampling times.

Table S3. Microbial counts (log cfu/g) and pH from the fermentative process of the vegetal models without and with proteolytic enzyme. Samples were taken at 0, 3, 8 and 15 days of incubation.

	Vegetal model without enzyme								Vegetal model with enzyme											
	**V0[1]**		**V3**		**V8**		**V15**		**VE0**		**VE3**		**VE8**		**VE15**		**RMSE[2]**	P_t[3]	P_E	P_{txE}
**PCA**	6.81	cd	7.62	bc	8.48	a	8.11	ab	6.8	d	8.2	ab	7.6	bcd	7.7	ab	0.29	***	ns	**
**LAB**	6.78	c	7.53	ab	7.88	ab	8.08	a	6.77	c	7.45	b	7.52	ab	7.64	ab	0.21	***	*	ns
**GC+**	5.84	de	6.51	cd	7.79	a	7.28	ab	5.67	e	6.65	bc	6.95	bc	7.13	abc	0.24	***	*	*
**YM**	6.69	ab	6.27	b	7.02	a	7.12	a	6.63	ab	6.67	ab	6.86	a	7.14	a	0.19	***	ns	ns
**pH**	6.78	a	5.83	b	5.23	c	4.74	d	6.61	a	4.47	de	4.32	e	4.21	e	0.12	***	***	***

[1]Vegetal models containing texturized pea protein without (V) and with (VE) enzyme at 0, 3, 8 and 15 d of incubation. [2]RMSE: root mean square of the errors. [3]*Pf*: *P* value of the time effect, P_E: *P* value of enzyme effect, P_{txE}: *P* value of interaction between time and enzyme effects. ***: $P < 0.001$; **: $P < 0.01$; *: $P < 0.5$; ns: $P > 0.05$. [4]Different letters in the same row indicate significant differences among models and sampling times.

Table S4. Free amino acids content (mg/g protein) in the animal model without and with proteolytic enzyme. Samples were taken at 0, 3, 8 and 15 days of incubation.

	Animal model without enzyme				Animal model with enzyme							
	A0[1]	A3	A8	A15	AE0	AE3	AE8	AE15	RMSE	*Pt*	*PE*	*P txE*
Asp	0.01 d	0.01 d	0.00 d	0.01 d	0.12 c	0.22 a	0.17 b	0.11 c	0.02	***	***	***
Glu	0.00	0.00	0.00	0.02	0.08	0.16	0.08	0.08	0.067	ns	**	ns
Ser	0.00 e	0.00 e	0.00 e	0.00 e	0.16 b	0.24 a	0.11 c	0.04 d	0.013	***	***	***
Asn	0.00 e	0.00 e	0.00 e	0.00 e	0.18 b	0.28 a	0.13 c	0.05 d	0.013	***	***	***
Gly	0.00 d	0.00 d	0.00 d	0.00 d	0.07 c	0.15 a	0.10 b	0.11 b	0.012	***	***	***
Gln	0.00 d	0.00 d	0.00 d	0.00 d	0.24 b	0.36 a	0.13 c	0.08 c	0.02	***	***	***
His	0.00	0.00	0.00	0.00	0.05	0.11	0.08	0.11	0.040	ns	***	ns
Thr	0.07 b	0.05 b	0.07 b	0.16 ab	0.24 ab	0.33 a	0.23 ab	0.15 ab	0.068	ns	***	**
Ala	0.00 c	0.00 c	0.00 c	0.02 c	0.22 b	0.58 a	0.41 a	0.48 a	0.063	***	***	**
Arg	0.00 b	0.00 b	0.00 b	0.00 b	0.36 a	0.34 a	0.00 b	0.15 ab	0.112	*	***	*
Pro	0.00 c	0.00 c	0.00 c	0.00 c	0.05 c	0.17 b	0.34 a	0.36 a	0.032	***	***	***
Tyr	0.00 c	0.00 c	0.00 c	0.00 c	0.26 b	0.38 a	0.00 c	0.00 c	0.022	***	***	***
Val	0.00 d	0.00 d	0.00 d	0.00 d	0.33 c	0.57 b	0.60 b	0.66 a	0.014	***	***	***
Met	0.00 c	0.00 c	0.00 c	0.03 bc	0.16 a	0.16 a	0.08 abc	0.09 ab	0.031	ns	***	*
Ile	0.00 d	0.00 c	0.00 d	0.00 d	0.37 c	0.65 b	0.71 a	0.72 a	0.018	***	***	***
Leu	0.00 d	0.00 c	0.00 d	0.00 d	0.69 c	1.13 a	1.04 b	1.07 b	0.019	***	***	***
Phe	0.00 b	0.00 b	0.00 b	0.00 b	0.49 a	0.61 a	0.51 a	0.41 a	0.071	ns	***	ns
Trp	0.00 b	0.00 b	0.00 b	0.00 b	0.03 ab	0.01 ab	0.00 b	0.27 a	0.090	ns	*	ns
Orn	0.00 b	0.00 b	0.00 b	0.00 b	0.00 b	0.06 ab	0.11 a	0.13 a	0.031	*	***	*
Lys	0.00 c	0.00 c	0.00 c	0.00 c	0.22 b	0.51 a	0.45 a	0.44 a	0.027	***	***	***
Total aac	0.08 d	0.06 d	0.08 d	0.23 d	4.31 c	7.00 a	5.27 b	5.50 b	0.189	***	***	***

[1]Animal models containing myofibrillar proteins without (A) and with (AE) enzyme at 0, 3, 8 and 15 d of incubation. [2]RMSE: root mean square of the errors. [3]*Pf*: *P* value of the time effect, P_E: *P* value of enzyme effect, P_{txE}: *P* value of interaction between time and enzyme effects. ***: $P < 0.001$; **: $P < 0.01$; *: $P < 0.5$; ns: $P > 0.05$. [4]Different letters in the same row indicate significant differences among models and times.

Table S5. Free amino acids content (mg/g protein) in the vegetal model without and with proteolytic enzyme. Samples were taken at 0, 3, 8 and 15 days of incubation.

	Vegetal model without enzyme								Vegetal model with enzyme											
	**V0**		**V3**		**V8**		**V15**		**VE0**		**VE3**		**VE8**		**VE15**		**RMSE**	**Pt**	**PE**	**PtxE**
Asp	0.03	b	0.06	b	0.06	b	0.04	b	0.06	b	0.36	a	0.53	a	0.60	a	0.086	***	***	***
Glu	0.45	c	0.36	c	0.47	c	0.45	c	0.60	c	3.17	b	4.28	a	5.07	a	0.320	***	***	***
Ser	0.00	d	0.00	d	0.01	d	0.01	d	0.36	d	1.79	c	2.38	b	2.84	a	0.147	***	***	***
Asn	0.18	e	0.03	e	0.03	e	0.01	e	0.59	d	1.25	c	1.61	b	2.00	a	0.086	***	***	***
Gly	0.01	d	0.03	d	0.05	d	0.03	d	0.10	d	0.68	c	1.08	b	1.59	a	0.058	***	***	***
Gln	0.00	c	0.02	c	0.04	c	0.01	c	0.80	b	1.85	a	2.03	a	2.05	a	0.081	***	***	***
His	0.00	b	0.01	b	0.02	b	0.07	b	0.14	b	0.51	a	0.55	a	0.59	a	0.077	***	***	**
Thr	0.14	d	0.13	d	0.28	d	0.14	d	0.36	d	1.38	c	2.29	b	3.11	a	0.140	***	***	***
Ala	0.03	d	0.03	d	0.08	d	0.08	d	0.56	d	3.09	c	3.96	b	4.73	a	0.254	***	***	***
Arg	0.00	d	0.00	d	0.00	d	0.00	d	0.92	c	2.19	a	2.05	ab	1.66	b	0.151	***	***	***
Pro	0.02	b	0.01	b	0.10	b	0.07	b	0.18	b	0.66	a	0.89	a	1.08	a	0.155	***	***	**
Tyr	0.01	b	0.00	b	0.02	b	0.00	b	0.62	b	4.55	a	5.52	a	5.04	a	0.356	***	***	***
Val	0.02	c	0.00	c	0.01	c	0.04	c	0.78	c	5.63	b	7.31	ab	9.21	a	0.857	***	***	***
Met	0.13	d	0.10	d	0.02	d	0.03	d	0.12	d	0.56	c	0.92	b	1.22	a	0.088	***	***	***
Ile	0.00	c	0.00	c	0.06	c	0.05	c	1.09	c	7.08	b	9.63	ab	12.51	a	1.024	***	***	***
Leu	0.02	d	0.01	d	0.02	d	0.06	d	2.72	d	17.00	c	24.06	b	30.61	a	1.773	***	***	***
Phe	0.03	d	0.01	d	0.09	d	0.04	d	1.84	d	11.17	c	16.91	b	22.11	a	1.493	***	***	***
Trp	0.03	c	0.02	c	0.06	c	0.08	c	0.22	c	0.71	b	1.05	b	1.53	a	0.119	***	***	***
Orn	0.00	c	0.07	bc	0.16	bc	0.18	bc	0.00	c	0.18	bc	0.49	ab	0.79	a	0.153	***	***	*
Lys	0.01	e	0.00	e	0.01	e	0.04	de	0.29	d	1.97	c	2.45	b	2.92	a	0.097	***	***	***
Total aac	1.11	d	0.88	d	1.57	d	1.45	d	12.35	d	65.78	c	89.98	b	111.25	a	6.553	***	***	***

[1] Vegetal models containing texturized pea protein without (V) and with (VE) enzyme at 0, 3, 8 and 15 d of incubation. [2]RMSE: root mean square of the errors. [3]*Pf*: *P* value of the time effect, P_E: *P* value of enzyme effect, P_{txE}: *P* value of interaction between time and enzyme effects. ***: $P < 0.001$; **: $P < 0.01$; *: $P < 0.5$; ns: $P > 0.05$. [4]Different letters in the same row indicate significant differences among models and times.

Table S6. Volatile compounds identified in the headspace of the animal and vegetal models. Animal models contain myofibrillar proteins without (A) and with (AE) enzyme. Vegetal models contain texturized pea protein without (V) and with (VE) enzyme.

	Compound	tr[1] (min)	LRI DB 624[2]	LRI std DB624[2]	RI[3]	Models A - AE	Models V - VE
	Aldehydes						
1	Acetaldehyde	2.13	469	466	a	s[4]	s
2	3-methylbutanal	11.02	690	687	a	s	s
3	2-methylbutanal	11.72	701	698	a	s	s
4	Pentanal	15.35	738	736	a	s	s
5	Hexanal	24.53	841	839	a	s	s
6	Heptanal	31.92	942	939	a	s	s
7	Octanal	39.41	1047	1044	a	s	s
8	Nonanal	46.27	1150	1148	a	s	s
9	Benzaldehyde	37.17	1017	1013	a	s	s
10	benzeneacetaldehyde	43.99	1109	1104	a	s	s
11	4-methyl-benzaldehyde	46.12	1148	-	b	s	s
12	2,4-dimethyl-benzaldehyde	52.79	1292	-	b	s	s
	Alcohols						
13	Ethanol	3.11	508	507	a	s	s
14	2-methyl-2-Propanol	4.62	569	-	b	s	s
15	3-methyl-3-Buten-1-ol	20.40	790	787	a	n	s
16	3-methyl-1-butanol	20.89	795	793	a	s	s
17	2-methyl-1-butanol	21.11	798	795	a	s	s
18	1-Pentanol	23.47	828	823	a	s	s
19	1-Hexanol	30.77	925	921	a	s	s
20	1-Heptanol	37.63	1024	1021	a	s	s
21	1-Octen-3-ol	38.12	1030	1028	a	s	s
22	2-Heptanol	32.40	949	947	a	s	s
23	Benzyl alcohol	44.70	1122	1120	a	s	s
24	1-Octanol	44.89	1126	1123	a	s	s
25	Phenylethyl alcohol	48.71	1195	1191	a	s	s
26	2-ethyl-1-Hexanol	42.21	1083	1083	a	s	s
	Ester compounds						
27	Methyl acetate	4.18	551	549	a	s	s
28	Ethyl Acetate	7.58	635	635	a	s	s
29	Methyl 3-methylbutirate	21.79	806	804	a	s	s
30	Butyl acetate	25.05	848	846	a	s	s
31	3-methyl-1-butanol acetate	29.56	907	905	a	n	s
	Alkanes						
32	Hexano	5.40	600	600	a	s	s
33	Toluene	20.18	788	790	a	s	s
34	Ethylbenzene	27.78	883	881	a	n	s
35	p-Xylene	28.42	891	893	a	s	s
36	o-xylene	30.24	917	915	a	s	s
37	Styrene	30.39	919	921	a	s	s

	Ketones						
38	Acetone	3.65	530	527	a	s	s
39	2,3-Butanedione	7.08	627	624	a	s	s
40	2-butanone	7.35	631	629	a	s	s
41	2-Pentanone	14.89	733	731	a	s	s
42	3-Pentanone	15.63	741	740	a	n	s
43	2-hexanone	24.12	836	835	a	s	s
44	3-heptanone	30.98	928	-	b	s	s
45	2-heptanone	31.49	936	933	a	s	s
46	4-methyl-2-Heptanone	34.56	981	-	b	s	s
47	2-Octanone	38.77	1038	1034	a	s	s
48	2-nonanone	45.79	1142	1139	a	s	s
49	3-Octen-2-one	43.13	1095	1094	a	s	s
50	2-pentyl-Furan	36.52	1009	1009	a	s	s
	Other compounds						
51	D-Limonene	39.15	1043	1046	a	s	s
52	Acetophenone	45.30	1133	1134	a	s	s
53	Isophorone	49.13	1203	1207	a	s	s
54	Terpinen-4-ol	50.18	1229	1228	a	s	s
	Pyrazines						
55	Pyrazine	18.63	772	772	a	n	s
56	Methyl-pyrazine (94)[5]	26.00	860	860	a	n	s
57	2,5-dimethyl-Pyrazine (108)	32.06	944	943	a	n	s
58	2-ethyl-5-methyl-pyrazine (121)	38.46	1034	1033	a	n	s
59	3-ethyl-2,5-dimethyl-pyrazine (135)	44.00	1109	1109	a	n	s
60	2,3-diethyl-5-methyl-Pyrazine (150)	48.08	1183	-	b	n	s
61	3,5-diethyl-2-methyl-pyrazine (150)	48.35	1188	-	b	n	s
62	3,5-diethyl-2-propyl-Pyrazine (122)	48.56	1192	-	b	n	s

[1]Tr: retention time, [2]LRI: Linear retention indices of the compounds (LRI DB624) or standards (LRI-std) eluted from GC-MS using a DB-624 capillary column. [3]Reliability of identification: a, identification by mass spectrum and by coincidence with the LRI of an authentic standard; b, tentatively identification by mass spectrum. [4](s) present in model, (n) absent in model. [5]Target ion (m/z in parenthesis) used to quantify the compound when the peak was not completely resolved.

Table S7. Volatile compounds content (AU 10^{-5}/g protein) in the headspace of the animal models (containing myofibrillar proteins) without and with proteolytic enzyme. Samples were taken at 0, 3, 8 and 15 days of incubation.

	Animal model without enzyme								Animal model with enzyme											
	A0[1]		A3		A8		A15		AE0		AE3		AE8		AE15		RMSE[2]	P_t[3]	P_E	P_{txE}
Aldehydes																				
Acetaldehyde	1.39		1.47		0.72		0.71		0.85		1.01		0.77		0.97		0.33	*ns*	*ns*	*ns*
3-methylbutanal	0.06	bc[4]	0.00	c	0.00	c	3.96	a	2.04	abc	3.79	a	3.05	abc	3.22	ab	0.93	*	***	*
2-methylbutanal	0.00	bc	0.00	b	0.43	ab	3.46	a	1.54	ab	3.25	a	2.79	a	3.00	a	0.70	*	***	*ns*
Pentanal	0.05	ab	0.00	b	0.23	ab	0.22	ab	0.00	b	0.24	ab	0.30	a	0.26	ab	0.06	*	*ns*	*ns*
Hexanal	1.30		2.49		2.25		1.70		1.29		1.03		1.10		1.10		0.65	*ns*	*	*ns*
Heptanal	0.23	b	0.29	ab	0.26	ab	0.59	a	0.16	b	0.22	b	0.47	ab	0.50	ab	0.10	**	*ns*	*ns*
Octanal	0.14		0.21		0.29		0.74		0.75		0.29		0.38		0.36		0.16	*ns*	*ns*	*ns*
Nonanal	0.58	b	0.82	b	1.34	b	2.64	a	1.02	b	0.45	b	2.66	a	2.66	a	0.76	**	*ns*	*ns*
Benzaldehyde	7.03	ab	3.61	b	3.68	b	5.16	ab	6.00	ab	5.19	ab	8.33	a	8.33	a	1.31	*ns*	*	*
Benzeneacetaldehyde	0.91		2.46		3.83		2.05		0.00		2.09		4.29		4.51		1.44	*ns*	*ns*	*ns*
4-methyl-benzaldehyde	0.90	c	2.53	abc	3.05	abc	5.10	ab	2.08	bc	2.98	abc	5.97	a	5.97	a	1.15	***	*	*ns*
2,4-dimethyl-benzaldehyde	1.20	b	1.03	b	1.32	ab	4.10	a	1.80	ab	2.17	ab	3.19	a	3.19	ab	0.85	**	*ns*	*ns*
Alcohols																				
Ethanol	6.78		5.12		7.61		4.28		6.54		5.78		10.46		9.56		6,15	ns	ns	ns
2-methyl-2-Propanol	0.00	e	0.23	c	0.17	d	0.29	b	0.31	a	0.19	d	0.30	ab	0.30	ab	0.00	***	***	***
3-methyl-3-Buten-1-ol																				
3-methyl-1-butanol	0.00	b	0.29	b	0.50	b	0.23	b	0.00	b	0.91	b	5.50	a	5.76	a	0.77	***	***	***
2-methyl-1-butanol	0.00	b	0.00	b	0.00	b	1.57	a	0.00	b	0.72	ab	1.56	a	1.63	a	0.26	***	***	**
1-Pentanol	0.00	b	0.00	b	0.00	b	0.01	b	0.00	b	0.32	a	0.00	b	0.00	b	0.02	***	***	***
1-Hexanol	1.50		0.64		0.50		0.78		0.39		3.93		3.61		3.52		1,36	*ns*	*	*ns*
1-Heptanol	0.00		0.00		6.99		3.55		5.27		29.25		12.18		9.85		10,48	*ns*	*	*ns*
1-Octen-3-ol	1.19	de	1.38	de	1.79	cd	3.07	ab	0.76	e	2.49	bc	3.38	a	3.38	a	0.26	***	***	***
2-Heptanol	0.44	ab	0.24	c	0.21	c	0.25	bc	0.27	bc	0.48	a	0.33	abc	0.33	abc	0.04	*ns*	*	**
Benzyl alcohol	0.00	b	0.48	b	0.81	b	1.96	a	0.00	b	0.00	b	2.28	a	2.40	a	0.22	***	**	**
1-Octanol	3.46	b	2.53	b	3.49	b	8.83	a	3.43	b	2.68	b	8.30	a	8.33	a	1.05	***	*ns*	**
Phenylethyl alcohol	0.00	b	0.00	b	0.21	b	4.59	a	0.00	b	0.00	b	6.48	a	6.57	a	0.53	***	***	***
2-ethyl-1-Hexanol	26.55		18.32		28.81		33.35		22.70		24.42		33.61		33.61		6.45	*	*ns*	*ns*
Esthers comp																				

Methyl acetate	0.33	b	0.49	ab	1.11	ab	1.10	ab	0.56	ab	0.35	b	1.42	a	1.42	a	0.30	***	*ns*	*ns*
Ethyl Acetate	5.55	c	24.84	a	1.10	c	0.65	c	5.18	c	13.16	b	0.57	c	0.57	c	1.71	***	***	***
Methyl 3-methylbutyrate	0.00	b	0.00	b	0.00	b	0.04	b	0.72	a	0.12	b	0.00	b	0.00	b	0.07	***	***	***
Butyl acetate	0.44	a	0.20	ab	0.17	b	0.24	ab	0.40	ab	0.18	ab	0.17	b	0.17	b	0.07	**	*ns*	*ns*
3-methyl-1-butanol acetate																				
Alkanes																				
Hexane	0.37		0.41		0.51		0.32		0.27		0.18		0.33		0.37		0.24	*ns*	*ns*	*ns*
Toluene	3.65	a	2.22	b	2.03	b	2.32	ab	4.09	a	3.11	ab	2.38	b	2.38	b	0.67	**	*ns*	*ns*
Ethylbenzene																				
p-Xylene	0.28		0.19		0.23		0.24		0.27		0.30		0.26		0.26		0.07	*ns*	*ns*	*ns*
o-xylene	0.15	a	0.09	b	0.13	a	0.17	a	0.14	a	0.00	b	0.18	a	0.17	a	0.02	***	*ns*	**
Styrene	0.25	b	0.00	d	0.11	c	0.30	b	0.57	a	0.49	a	0.21	bc	0.21	bc	0.01	***	***	***
Ketones																				
Acetone	0.23	b	1.65	b	0.76	b	4.85	a	0.25	b	1.24	b	4.45	a	4.45	a	0.49	***	**	***
2,3-Butanedione	0.63		0.80		0.56		0.67		0.64		0.79		0.72		0.72		0.23	*ns*	*ns*	*ns*
2-butanone	0.70	d	1.21	cd	0.80	d	2.03	ab	1.07	cd	1.62	bc	2.20	a	2.20	a	0.17	***	***	***
2-Pentanone	0.00	c	0.84	ab	0.42	bc	1.16	a	0.00	c	0.90	ab	1.22	a	1.23	a	0.15	***	**	**
3-Pentanone																				
2-hexanone	0.27		1.17		1.03		0.39		0.62		0.22		0.30		0.29		0.40	*ns*	*ns*	*ns*
3-heptanone	0.89		1.03		0.51		0.00		0.37		0.75		0.12		0.17		0.33	*ns*	*ns*	*ns*
2-heptanone	0.46	ab	0.41	b	0.40	b	0.75	a	0.51	ab	0.40	b	0.55	ab	0.55	ab	0.10	*	*ns*	*ns*
4-methyl-2-Heptanone	0.43	b	0.69	ab	0.45	b	0.83	a	0.40	b	0.97	a	0.87	ab	0.93	a	0.19	*	*ns*	*ns*
2-Octanone	0.42	ab	0.27	b	0.27	b	0.46	a	0.35	ab	0.28	b	0.45	a	0.46	a	0.05	**	*ns*	*
2-nonanone	0.81	b	0.83	b	2.43	ab	4.73	a	2.51	ab	0.83	b	3.68	a	3.68	a	1.40	*	*ns*	*ns*
3-Octen-2-one	0.00	b	0.75	ab	1.42	ab	1.81	a	0.70	ab	0.16	b	1.28	ab	1.39	ab	0.47	*	*ns*	*ns*
Other compounds																				
2-pentyl-Furan	0.00	b	0.00	b	0.00	b	0.00	b	0.00	b	0.00	b	0.16	a	0.14	a	0.03	**	***	**
D-Limonene	0.26	b	0.61	b	1.07	b	0.00	b	0.76	b	2.11	ab	4.47	a	4.63	a	0.87	*	***	*ns*
Acetophenone	4.16	ab	3.47	b	3.82	b	6.70	a	5.54	ab	3.26	b	6.53	a	6.53	a	1.42	*	*ns*	*ns*
Isophorone	0.84	a	0.00	c	0.00	c	0.00	c	0.85	a	0.28	b	0.00	c	0.00	c	0.07	***	*ns*	*ns*
Terpinen-4-ol	0.00		0.52		0.76		3.68		0.00		0.95		1.47		1.32		1.00	*ns*	*ns*	*ns*

[1]Animal models containing myofibrillar proteins without (A) and with (AE) enzyme at 0, 3, 8 and 15 d of incubation. [2]RMSE: root mean square of the errors. [3]*Pf*: *P* value of the time effect, P_E: *P* value of enzyme effect, P_{txE}: *P* value of interaction between time and enzyme effects. ***: $P < 0.001$; **: $P < 0.01$; *: $P < 0.5$; ns: $P > 0.05$. [4]Different letters in the same row indicate significant differences among models and times.

Table S8. Evolution of the content of volatile compounds (AU 10^{-5}/g protein) in the headspace of vegetal models (containing pea protein) without and with proteolytic enzyme. Samples were taken at 0, 3, 8 and 15 days of incubation.

	Vegetal model without enzyme								Vegetal model with enzyme											
	V0[1]		V3		V8		V15		VE0		VE3		VE8		VE15		RMSE[2]	Pt[3]	PE	P txE
**Aldehydes**																				
Acetaldehyde	0.47		0.45		0.44		0.37		0.26		0.24		0.21		0.27		0.13	ns	**	ns
3-methylbutanal	0.22	c[4]	0.00	d	0.00	d	0.00	d	0.37	c	0.86	a	0.40	c	0.59	b	0.06	***	***	***
2-methylbutanal	0.29	c	0.00	d	0.00	d	0.00	d	0.40	c	0.69	a	0.37	c	0.53	b	0.04	***	***	***
Pentanal	3.18	a	3.37	a	1.57	b	0.73	bc	1.10	bc	0.29	c	0.21	c	0.58	bc	0.38	***	***	***
Hexanal	24.53	a	25.01	a	29.98	a	29.20	a	19.43	ab	6.91	bc	1.06	c	1.72	c	5.32	ns	***	*
Heptanal	0.93	abc	1.16	a	0.88	abc	1.11	ab	0.55	bc	0.56	abc	0.44	c	0.77	abc	0.21	ns	***	ns
Octanal	1.19	abc	1.38	ab	0.86	abc	1.57	a	0.52	bc	0.41	c	0.43	bc	0.96	abc	0.33	*	***	ns
Nonanal	7.69	a	7.30	a	2.32	b	1.83	b	1.02	b	1.39	b	1.21	b	1.76	b	1.53	*	***	**
Benzaldehyde	8.25	abc	10.78	a	7.22	bcd	6.74	bcde	9.69	ab	4.97	cde	3.43	e	4.35	ed	1.19	***	***	***
Benzeneacetaldehyde	1.32		0.00		0.00		0.00		0.00		0.00		0.00		0.00		0.44	ns	ns	ns
4-methyl-benzaldehyde	0.35	c	0.89	a	0.87	a	0.71	ab	0.30	c	0.43	bc	0.31	c	0.61	abc	0.11	**	***	**
2,4-dimethyl-benzaldehyde	0.37	b	1.85	a	0.79	b	0.80	b	0.64	b	0.30	b	0.31	b	0.32	b	0.33	*	**	**
**Alcohols**																				
Ethanol	0.60		1.67		15.21		55.14		0.70		4.91		2.53		3.17		21.82	ns	ns	ns
2-methyl-2-Propanol	0.00	b	0.16	ab	0.16	ab	0.27	a	0.00	b	0.28	a	0.18	ab	0.26	a	0.06	***	ns	ns
3-methyl-3-Buten-1-ol	0.59	cd	2.23	b	0.00	d	0.27	d	0.00	d	1.93	bc	3.88	a	5.17	a	0.39	***	***	***
3-methyl-1-butanol	0.00	c	0.98	c	1.70	c	4.43	bc	0.36	c	8.70	bc	14.94	ab	24.06	a	4.56	**	***	*
2-methyl-1-butanol	0.00	c	0.00	c	0.17	c	0.46	bc	0.00	c	1.05	bc	2.40	ab	3.84	a	0.70	**	***	**
1-Pentanol	2.08		0.00		1.17		3.12		2.13		2.71		2.32		2.41		1.12	ns	*	ns
1-Hexanol	0.98	f	5.17	e	9.25	d	11.45	c	2.58	f	16.55	b	17.64	b	27.36	a	0.69	***	***	***
1-Heptanol	2.84	bc	14.70	a	12.86	a	4.69	b	3.27	bc	2.89	bc	1.58	c	2.03	bc	0.85	***	***	***
1-Octen-3-ol	2.04	d	2.84	cd	4.22	abc	4.76	ab	3.42	bcd	3.16	bcd	2.91	cd	5.74	a	0.58	***	ns	**
2-Heptanol	0.34	b	0.57	b	4.95	ab	6.77	a	0.36	b	0.68	b	1.08	b	2.21	ab	1.60	*	*	ns
Benzyl alcohol	0.62	e	1.12	cde	1.50	bcd	1.81	ab	1.01	de	1.45	bcd	1.59	abc	2.13	a	0.19	***	**	ns
1-Octanol	2.14	bc	2.81	bc	3.21	ab	4.46	a	2.07	bc	2.19	bc	1.73	c	2.30	bc	0.46	**	***	**
Phenylethyl alcohol	0.00	f	0.00	f	0.02	ef	0.86	d	0.33	e	2.46	c	6.63	b	12.93	a	0.13	***	***	***
2-ethyl-1-Hexanol	25.69	bc	28.77	bc	34.78	b	54.60	a	17.75	c	18.65	c	25.73	bc	28.42	bc	4.06	***	***	**
**Esthers comp**	**2.31**		**4.41**		**2.71**		**4.33**		**2.61**		**3.30**		**2.11**		**2.42**					

Methyl acetate	1.13	ab	1.27	a	0.69	b	1.07	ab	1.32	a	0.89	ab	0.71	b	0.72	b	0.19	**	ns	*
Ethyl Acetate	0.99		2.12		1.25		2.26		0.81		1.31		0.42		0.00		0.76	ns	*	ns
Methyl 3-methylbutyrate	0.00	c	0.27	b	0.00	c	0.00	c	0.00	c	0.35	b	0.37	b	0.66	a	0.06	***	***	***
Butyl acetate	0.20	d	0.41	b	0.30	bcd	0.73	a	0.25	d	0.26	cd	0.27	cd	0.39	bc	0.05	***	***	***
3-methyl-1-butanol acetate	0.00	e	0.33	bcd	0.48	abc	0.27	cd	0.24	d	0.50	ab	0.35	bcd	0.65	a	0.07	***	***	***
**Alkanes**																				
Hexane	0.19		0.33		0.10		0.19		0.00		0.27		0.41		0.49		0.19	ns	ns	*
Toluene	3.94		3.52		4.08		2.44		4.14		1.41		1.41		2.39		0.85	*	**	*
Ethylbenzene	0.10	ab	0.22	a	0.17	ab	0.14	ab	0.00	b	0.00	b	0.00	b	0.00	b	0.06	ns	***	ns
p-Xylene	0.39	a	0.41	a	0.28	ab	0.29	ab	0.24	ab	0.19	b	0.24	ab	0.22	b	0.05	ns	***	ns
o-xylene	0.20	a	0.21	a	0.23	a	0.18	a	0.18	a	0.14	a	0.00	b	0.17	a	0.04	*	***	***
Styrene	0.89	b	0.51	b	1.06	b	1.27	b	4.12	a	0.86	b	1.03	b	1.31	b	0.64	**	**	**
**Ketones**																				
Acetone	1.82	b	3.22	b	2.15	b	2.88	b	2.00	b	3.32	b	4.32	b	8.58	a	1.17	***	***	***
2,3-Butanedione	0.46		2.14		0.95		0.27		1.10		0.35		0.36		0.70		0.70	ns	ns	*
2-butanone	2.95	ab	3.79	ab	2.33	ab	1.15	b	2.69	ab	3.55	ab	3.63	ab	6.18	a	1.46	ns	*	*
2-Pentanone	0.41	b	0.91	b	0.51	b	0.71	b	0.59	b	1.21	b	1.37	b	2.93	a	0.50	***	***	**
3-Pentanone	0.24	b	0.24	b	0.26	b	0.32	b	0.28	b	0.36	b	0.45	b	1.61	a	0.26	***	***	***
2-hexanone	1.72	bc	1.91	abc	1.03	c	0.93	c	1.89	abc	2.14	ab	2.39	ab	2.87	a	0.42	ns	***	**
3-heptanone	0.34	b	5.58	a	1.76	ab	1.17	b	0.46	b	0.00	b	0.00	b	0.00	b	1.45	*	**	*
2-heptanone	15.95	a	13.37	ab	10.64	ab	4.93	b	18.92	a	13.33	ab	12.73	ab	15.27	ab	3.37	*	*	ns
4-methyl-2-Heptanone	0.29	e	0.89	a	0.60	bc	1.05	a	0.29	e	0.50	cd	0.36	de	0.70	b	0.08	***	***	***
2-Octanone	1.09		1.14		0.94		0.79		0.94		0.75		0.67		1.05		0.19	ns	ns	*
2-nonanone	0.96	b	0.90	b	0.96	b	1.14	a	0.70	b	0.70	b	0.67	b	1.10	a	0.17	*	*	ns
3-Octen-2-one	0.76	b	0.77	b	0.96	b	1.28	a	0.52	c	0.52	c	0.41	c	0.47	c	0.10	***	***	***
**Other compounds**																				
2-pentyl-Furan	0.40	c	0.64	bc	1.08	a	0.84	ab	0.46	bc	1.05	a	0.50	bc	0.78	abc	0.13	***	ns	***
D-Limonene	0.52	ab	0.28	b	0.31	b	0.91	a	0.23	b	0.37	b	0.29	b	0.44	ab	0.17	*	*	ns
Acetophenone	2.63	b	3.49	b	3.03	b	4.79	a	2.62	b	2.71	b	2.63	b	2.73	b	0.41	***	***	***
Isophorone	0.26	ab	0.38	ab	0.31	ab	0.34	ab	0.17	b	0.32	ab	0.42	ab	0.54	a	0.12	*	ns	ns
Terpinen-4-ol	0.27	d	0.24	d	0.19	d	0.48	c	0.30	d	0.74	b	0.88	ab	0.99	a	0.11	***	***	***
**Pyrazines**																				
Pyrazine	0.00	b	0.31	ab	0.23	ab	0.11	b	0.91	a	0.40	ab	0.00	b	0.26	ab	0.23	ns	*	*
methyl-pyrazine (94)	0.29		0.29		0.30		0.33		0.30		0.32		0.32		0.36		0.04	ns	ns	ns

2,5-dimethyl-Pyrazine (108)	1.28		1.41		1.51		1.30		1.38		1.37		1.61		3.31		0.78	ns	ns	ns
2-ethyl-5-methyl-pyrazine (121)	0.58		0.60		0.67		0.56		0.67		0.58		0.55		0.64		0.08	ns	ns	ns
3-ethyl-2,5-dimethyl-pyrazine (135)	1.68		2.12		2.58		2.22		2.76		2.28		2.14		2.45		0.40	ns	ns	*
2,3-diethyl-5-methyl-Pyrazine (150)	0.12	ab	0.10	b	0.11	ab	0.11	b	0.14	a	0.11	ab	0.10	b	0.12	ab	0.01	*	ns	ns
3,5-diethyl-2-methyl-pyrazine (150)	0.21	abc	0.17	c	0.21	abc	0.20	bc	0.27	a	0.23	abc	0.22	abc	0.25	ab	0.02	ns	***	ns
3,5-diethyl-2-propyl-Pyrazine (122)	0.10	ab	0.00	c	0.08	b	0.09	b	0.12	a	0.08	b	0.00	c	0.10	ab	0.02	***	*	***

[1] Vegetal models containing pea protein without (V) and with (VE) enzyme at 0, 3, 8 and 15 d of incubation. [2]RMSE: root mean square of the errors. [3]*Pf*: *P* value of the time effect, P_E: *P* value of enzyme effect, P_{txE}: *P* value of interaction between time and enzyme effects. ***: *P* < 0.001; **: *P* < 0.01; *: *P* < 0.5; ns: *P* > 0.05. [4]Different letters in the same row indicate significant differences among models and times.
